# Supplementary material for: Liver stiffness regression after sustained virological response by direct-acting antivirals reduces the risk of outcomes
Source: Sci Rep. 2021 Jun 3;11:11681. doi: 10.1038/s41598-021-91099-1 (PMC8175552; doi:10.1038/s41598-021-91099-1)
Supplement: Supplementary file 1 — Supplementary Information. [file 41598_2021_91099_MOESM1_ESM.docx]

**Supplementary Material**

**Liver stiffness regression after sustained virological response by direct-acting antivirals reduces the risk of outcomes**

Juliana Piedade^1,2^; Gustavo Pereira^2,3^; Lívia Guimarães^2^; Joana Duarte^2^; Lívia Victor^2^; Caroline Baldin^2^; Cintia Inacio^1^; Ricardo Santos^1^; Úrsula Chaves^1^; Estevão P Nunes^1^; Beatriz Grinsztejn^1^; Valdilea G Veloso^1^, Flavia Fernandes^2^; Hugo Perazzo^1^*

**Institutional affiliations**

^1^ Oswaldo Cruz Foundation (FIOCRUZ), Evandro Chagas National Institute of Infectious Diseases (INI), Rio de Janeiro, Brazil

^2^ Bonsucesso Federal Hospital, Hepatology Department, Rio de Janeiro, Brazil

^3^ Estacio de Sa University, School of Medicine, Rio de Janeiro, Brazil

|  | **Page** |
| --- | --- |
| **Supplementary Table 1.** Multivariate Cox models to predict incidence of liver related complications or death during follow-up after sustained virological response in patients with unfavourable Baveno VI status (LSM ≥ 20 kPa or platelet count < 150 x 10^9^/ mm^3^) | **2** |
| **Supplementary Table 2.** Factors associated with incidence of clinical outcomes during follow-up after sustained virological response considering the liver stiffness measurement performed more distant from the end of HCV treatment [median time = 12.5 months (IQR, 10.3-14.9)] | **3** |
| **Supplementary Table 3.** Factors associated with incidence of liver related complications or death during follow-up after sustained virological response in patients with HCV mono-infection [n=431 patients and 27 outcomes] | **4** |
| **Supplementary Table 4.** Sensitivity analyses to identify factors associated with incidence of liver related complications or death during follow-up after sustained virological response in patients with HCV mono-infection considering only those patients with unfavorable Baveno VI criteria [n=236 patients and 24 outcomes] or all HCV-infected individuals and liver stiffness measurement (LSM) performed more distant from the end of HCV treatment [n=431 patients and 27 outcomes]. | **5** |
| **Appendix I.** STROBE checklist of the study entitled “**Liver stiffness regression after sustained virological response by direct-acting antivirals reduces the risk of outcomes**” | **6** |

**Supplementary Table 1.** Multivariate Cox models to predict incidence of liver related complications or death during follow-up after sustained virological response in patients with unfavourable Baveno VI status (LSM ≥ 20 kPa or platelet count < 150 x 10^9^/ mm^3^)

|  | **Model A** | | **Model B** | |
| --- | --- | --- | --- | --- |
|  | **HR [95% CI]** | **p value** | **HR [95% CI]** | **p value** |
| Male gender (vs female) | 2.59 [1.16-5.78] | 0.021 | 3.16 [1.27-7.89] | 0.014 |
| Age (per 10 years) | 1.02 [0.98-1.06] | 0.389 | 1.00 [0.96-1.05] | 0.940 |
| AST (per 10 UI/L) |  |  | 0.98 [0.90-1.06] | 0.558 |
| Albumin level < 3.5 mg/dL (vs ≥ 3.5 mg/dL) |  |  | 5.56 [2.22-13.97] | < 0.001 |
| Regression of LSM ≥ 20% after SVR (vs < 20%) | 0.37 [0.16-0.88] | 0.025 | 0.31 [0.12-0.80] | 0.016 |

A time-dependent multivariate Cox models adjusted for age and gender (Model A) and adjusted for variables found to be associated (p value ≤ 0.10) with LRC or death in the univariate analysis (Model B). Liver-related complications were ascites, hepatic encephalopathy, variceal bleeding or hepatocellular carcinoma. AST, aspartate aminotransferase; CI, confidence interval; HR, hazard ratio; LSM, liver stiffness measurement; SVR, sustained virological response

**Supplementary Table 2.** Factors associated with incidence of clinical outcomes during follow-up after sustained virological response considering the liver stiffness measurement performed more distant from the end of HCV treatment [median time = 12.5 months (IQR, 10.3-14.9)]

|  | **Multivariate analysis** | |
| --- | --- | --- |
|  | **HR [95% CI]** | **p value** |
| Male gender (vs female) | 2.96 [1.29-6.82] | 0.011 |
| Age (per 10 years) | 1.00 [0.99-1.01] | 0.615 |
| AST (per 10 UI/L) | 0.98 [0.91-1.05] | 0.581 |
| Albumin level < 3.5 mg/dL (vs ≥ 3.5 mg/dL) | 4.33 [1.89-9.93] | 0.001 |
| Baveno VI status (unfavorable vs favorable) | 4.72 [1.32-16.80] | 0.017 |
| Regression of LSM ≥ 20% after SVR (vs < 20%) | 0.38 [0.17-0.86] | 0.021 |

A time-dependent Cox model was used for the analysis adjusted age, gender and all variables found to be associated (p value ≤ 0.10) with LRC or death in the univariate analysis were entered into the multivariate Cox models. The variable Baveno VI status (favorable vs. unfavorable) replaced the variables LSM and platelet count at baseline in the models due to collinearity. Baveno VI status was defined as unfavorable if LSM ≥ 20 kPa or platelet count < 150 x 10^9^/ mm^3^. Liver-related complications were ascites, hepatic encephalopathy, variceal bleeding or hepatocellular carcinoma.AST, aspartate aminotransferase; CI, confidence interval; HR, hazard ratio; LSM, liver stiffness measurement; SVR, sustained virological response.

**Supplementary Table 3.** Factors associated with incidence of liver related complications or death during follow-up after sustained virological response in patients with HCV mono-infection [n=431 patients and 27 outcomes]

|  | **Univariate analysis** | | **Multivariate analysis** | |
| --- | --- | --- | --- | --- |
|  | **HR [95% CI]** | **p value** | **HR [95% CI]** | **p value** |
| Male gender (vs female) | 2.24 [1.05-4.76] | 0.037 | 2.92 [1.29-6.65] | 0.010 |
| Age (per 10 years) | 1.01 [0.94-1.08] | 0.832 | 1.00 [0.99-1.01] | 0.740 |
| Type-2 diabetes (yes vs no) | 0.98 [0.88-1.09] | 0.757 |  |  |
| Hypertension (yes vs no) | 0.90 [0.42-1.93] | 0.787 |  |  |
| Dyslipidemia (yes vs no) | 1.00 [0.98-1.02] | 0.810 |  |  |
| HCV genotype-1 (vs other) | 0.53 [0.20-1.39] | 0.196 |  |  |
| Experimented patients (vs naive) | 1.48 [0.69-3.20] | 0.317 |  |  |
| SOF/DCV regimen (vs others) | 2.23 [0.84-5.90] | 0.106 |  |  |
| ALT (per 10 UI/L) | 1.01 [0.96-1.07] | 0.668 |  |  |
| AST (per 10 UI/L) | 1.04 [0.99-1.10] | 0.148 |  |  |
| Albumin level < 3.5 mg/dL (vs ≥ 3.5 mg/dL) | 4.49 [2.04-9.86] | < 0.001 | 4.29 [1.88-9.81] | 0.001 |
| Baveno VI status (unfavorable vs favorable) | 6.20 [1.87-20.60 | 0.003 | 4.00 [1.17-13.66] | 0.027 |
| Regression of LSM ≥ 20% after SVR (vs < 20%) | 0.48 [0.22-1.03] | 0.060 | 0.48 [0.21-1.08] | 0.077 |

A time-dependent Cox model was used for the analysis. Variables found to be associated (p value ≤ 0.10) with LRC or death in the univariate analysis were entered into the multivariate Cox models adjusted for age and gender. Baveno VI status was defined as unfavorable if LSM ≥ 20 kPa or platelet count < 150 x 10^9^/ mm^3^. ALT, alanine aminotransferase; AST, aspartate aminotransferase; CI, confidence interval; HR, hazard ratio; LSM, liver stiffness measurement; SOF, sofosbuvir; DCV, daclatasvir

**Supplementary Table 4.** Sensitivity analyses to identify factors associated with incidence of liver related complications or death during follow-up after sustained virological response in patients with HCV mono-infection considering only those patients with unfavorable Baveno VI criteria [n=236 patients and 24 outcomes] or all HCV-infected individuals and liver stiffness measurement (LSM) performed more distant from the end of HCV treatment [n=431 patients and 27 outcomes].

|  | **HCV mono-infected patients with unfavorable Baveno VI criteria & LSM post-SVR closest to the end-of-treatment [n=236 patients and 24 outcomes]** | | | | **All HCV mono-infected patients & LSM post-SVR more distant to the end-of-treatment**  **[n=431 patients and 27 outcomes]** | | | |
| --- | --- | --- | --- | --- | --- | --- | --- | --- |
|  | **Univariate analysis** | | **Multivariate analysis** | | **Univariate analysis** | | **Multivariate analysis** | |
|  | **HR [95% CI]** | **p value** | **HR [95% CI]** | **p value** | **HR [95% CI]** | **p value** | **HR [95% CI]** | **p value** |
| Male gender (vs female) | 2.16 [0.97-4.81] | 0.059 | 3.01 [1.23-7.34] | 0.015 | 2.24 [1.05-4.76] | 0.037 | 2.91 [1.28-6.60] | 0.011 |
| Age (per 10 years) | 1.26 [0.81-1.95] | 0.302 | 1.00 [0.95-1.05] | 0.998 | 1.01 [0.94-1.08] | 0.832 | 1.00 [0.99-1.01] | 0.650 |
| Type-2 diabetes (yes vs no) | 0.98 [0.89-1.09] | 0.769 |  |  | 0.98 [0.88-1.09] | 0.757 |  |  |
| Hypertension (yes vs no) | 0.96 [0.79-1.18] | 0.720 |  |  | 0.90 [0.42-1.93] | 0.787 |  |  |
| Dyslipidemia (yes vs no) | 1.00 [0.98-1.02] | 1.000 |  |  | 1.00 [0.98-1.02] | 0.810 |  |  |
| HCV genotype-1 (vs other) | 0.56 [0.19-1.64] | 0.294 |  |  | 0.53 [0.20-1.39] | 0.196 |  |  |
| Experimented patients (vs naive) | 1.29 [0.57-2.90] | 0.541 |  |  | 1.48 [0.69-3.20] | 0.317 |  |  |
| SOF/DCV regimen (vs others) | 2.56 [0.76-8.57] | 0.129 |  |  | 2.23 [0.84-5.90] | 0.106 |  |  |
| ALT (per 10 UI/L) | 0.99 [0.94-1.05] | 0.794 |  |  | 1.01 [0.96-1.07] | 0.668 |  |  |
| AST (per 10 UI/L) | 1.01 [0.95-1.08] | 0.717 |  |  | 1.04 [0.99-1.10] | 0.148 |  |  |
| Albumin level < 3.5 mg/dL (vs ≥ 3.5 mg/dL) | 3.90 [1.67-9.14] | 0.002 | 5.19 [2.11-12.79] | < 0.001 | 4.49 [2.04-9.86] | < 0.001 | 4.17 [1.83-9.47] | 0.001 |
| Baveno VI status (unfavorable vs favorable) | NA | NA |  |  | 6.20 [1.87-20.60 | 0.003 | 4.06 [1.19-13.90] | 0.026 |
| Regression of LSM ≥ 20% after SVR (vs < 20%) | 0.42 [0.18-0.97] | 0.043 | 0.33 [0.13-0.84] | 0.021 | 0.36 [0.16-0.78] | 0.010 | 0.39 [0.17-0.89] | 0.025 |

A time-dependent Cox model was used for the analyses. Variables found to be associated (p value ≤ 0.10) with liver-related complications or death in the univariate analysis were entered into the multivariate Cox models adjusted for age and gender. Baveno VI status was defined as unfavorable if LSM ≥ 20 kPa or platelet count < 150 x 10^9^/ mm^3^. ALT, alanine aminotransferase; AST, aspartate aminotransferase; CI, confidence interval; HR, hazard ratio; LSM, liver stiffness measurement; SOF, sofosbuvir; DCV, daclatasvir

**Appendix I.** STROBE checklist of the study entitled “**Liver stiffness regression after sustained virological response by direct-acting antivirals reduces the risk of outcomes**”

STROBE Statement—Checklist of items that should be included in reports of ***cohort studies***

|  | Item No | Recommendation |
| --- | --- | --- |
| **Title and abstract** | 1 | (*a*) Indicate the study’s design with a commonly used term in the title or the abstract  **Abstract; page ii** |
|  |  | (*b*) Provide in the abstract an informative and balanced summary of what was done and what was found  **Abstract; page ii** |
| Introduction | | |
| Background/rationale | 2 | Explain the scientific background and rationale for the investigation being reported  **Page 3 (1-30)**  Direct-acting antivirals (DAA) have changed the natural history of chronic hepatitis C in the last decade.^1^ These regimens are safe and highly effective, leading to rates of sustained virological response (SVR) higher than 90%.^2,3^ Therefore, international guidelines were updated to recommend DAA treatment to all individuals with chronic hepatitis C.^4,5^ A recent large cohort study demonstrated that treatment with DAAs is associated with reduced risk for mortality and hepatocellular carcinoma (HCC), reinforcing the long-term impact of SVR.^6^ However, the burden of liver-related events in patients with hepatitis C virus (HCV) that have successfully cleared the virus persists, particularly in individuals with cirrhosis previous to treatment.^7^  Historically, liver biopsy remains the reference for liver fibrosis staging and detection of cirrhosis. However, this invasive method has been challenged by several limitations and performing a liver biopsy in SVR patients without abnormal liver tests might not be justified. Liver stiffness measurement (LSM) by transient elastography (TE) is the most validated non-invasive method to assess liver fibrosis in patients with ongoing HCV infection as an alternative to liver biopsy.^8^ LSM by TE can be used to predict severe outcomes in chronic hepatitis C: patients with LSM ≥ 9.5 kPa (advanced fibrosis/cirrhosis) had significantly lower overall survival compared to those in the lower range.^9^ Additionally, combining LSM values with platelet count in HCV-infected patients was extensively validated to identify high-risk esophageal varices.^10^  Despite the broad validation of the diagnostic and prognostic value of LSM in patients with HCV, the correct interpretation of LSM in patients after SVR remains unclear. Several recent studies have reported a significant regression of LSM after SVR in patients with HCV treated by DAAs.^11,12^ However, it is still controversial whether the decrease of LSM after HCV eradication is related to suppression of viral necro-inflammatory activity rather than regression of liver fibrosis. Moreover, the extent of the long-term clinical impact of LSM reduction after SVR in patients with HCV treated by DAAs remains unclear because most trials had short follow-up periods and such severe outcomes are rare. Establishing an accurate longitudinal non-invasive strategy which could be used to predict severe outcomes after SVR would be clinically and epidemiologically important. The primary aim of this study was to evaluate the prognostic value of LSM regression after SVR by DAAs. The secondary aim was to identify risk factors associated with liver related complications (LRC) or death in patients with HCV after SVR. |
| Objectives | 3 | State specific objectives, including any prespecified hypotheses  **Page 3 (27-30)**  The primary aim of this study was to evaluate the prognostic value of LSM regression after SVR by DAAs. The secondary aim was to identify risk factors associated with liver related complications (LRC) or death in patients with HCV after SVR. |
| Methods | | |
| Study design | 4 | Present key elements of study design early in the paper  **Page 3 (34-35)**  This retrospective observational study was conducted at two tertiary centers for management of patients with viral hepatitis in Rio de Janeiro, Brazil. |
| Setting | 5 | Describe the setting, locations, and relevant dates, including periods of recruitment, exposure, follow-up, and data collection  **Page 3 (34-36)**  This retrospective observational study was conducted at two tertiary centers for management of patients with viral hepatitis in Rio de Janeiro, Brazil. All adult patients with chronic hepatitis C treated by DAAs between October 2015 and November 2019 were eligible. |
| Participants | 6 | (*a*) Give the eligibility criteria, and the sources and methods of selection of participants. Describe methods of follow-up  **Pages 3/4 (35-40)**  All adult patients with chronic hepatitis C treated by DAAs between October 2015 and November 2019 were eligible. The exclusion criteria were: (1) HCV treatment failure, loss of follow-up or missing data of SVR; (2) presence of hepatitis B coinfection, liver transplantation or hepatic decompensation (ascites, hepatic encephalopathy, variceal bleeding or HCC) before HCV treatment, (3) non-available LSM before or after DAA and (4) LSM < 10 kPa before HCV treatment.  **Page 4(42-43)**  HCC screening and esophageal varices surveillance were performed according to the current international guidelines^4,5,13^  **Page 4 (52-73)**  Clinical features included comorbidities, data of HCV history/treatment and liver stiffness by TE before and after DAA therapy. Laboratory parameters (including liver enzymes, albumin, creatinine, fasting glucose and platelet count) performed at least three months before or after each LSM were collected. All medical records from included patients were reviewed by trained investigators to identify the prospective incidence of the following primary outcomes after SVR: LRC (ascites, hepatic encephalopathy, variceal bleeding, or HCC), liver transplantation or death.  ***Liver stiffness measurement***  TE examinations were performed before and after HCV treatment in patients with a 3-h fasting by experienced operators. Liver fibrosis staging by TE was considered as reliable for analysis if the following criteria had been met: (1) at least 10 valid measurements; (2) an interquartile range (IQR) lower than 30% of the median of LSM and (3) a success rate of more than 60%. TE examination obtained closest to the date of start and the end of DAA treatment was considered for LSM before treatment and after SVR, respectively. Patients with LSM ≥ 10 kPa before HCV treatment were classified as compensated advanced chronic liver disease (c-ACLD). *l*. Additionally, those patients with LSM ≥ 20 kPa or platelet count < 150 × 10^9^/mm^3^ before HCV treatment were considered as having an unfavorable Baveno VI status.^13^ |
|  |  | (*b*) For matched studies, give matching criteria and number of exposed and unexposed  **Not applicable** |
| Variables | 7 | Clearly define all outcomes, exposures, predictors, potential confounders, and effect modifiers. Give diagnostic criteria, if applicable  **Page 4 (40-41)**  SVR was defined as undetectable HCV RNA at 12 weeks following the conclusion of treatment.  **Page 4 (55-58)**  All medical records from included patients were reviewed by trained investigators to identify the prospective incidence of the following primary outcomes after SVR: LRC (ascites, hepatic encephalopathy, variceal bleeding, or HCC ), liver transplantation or death.  **Page 4 (66-69)**  TE examination obtained closest to the date of start and the end of DAA treatment was considered for LSM before treatment and after SVR, respectively. Patients with LSM ≥ 10 kPa before HCV treatment were classified as compensated advanced chronic liver disease (c-ACLD). |
| Data sources/ measurement | 8* | For each variable of interest, give sources of data and details of methods of assessment (measurement). Describe comparability of assessment methods if there is more than one group  **Pages 4 (49-73)**  ***Data collection***  Demographic, clinical and biological data were collected by trained investigators using an electronic case-report form at Research Electronic Data Capture (REDCap, https://projectredcap.org/). Clinical features included comorbidities, data of HCV history/treatment and liver stiffness by TE before and after DAA therapy. Laboratory parameters (including liver enzymes, albumin, creatinine, fasting glucose and platelet count) performed at least three months before or after each LSM were collected. All medical records from included patients were reviewed by trained investigators to identify the prospective incidence of the following primary outcomes after SVR: LRC (ascites, hepatic encephalopathy, variceal bleeding, or HCC), liver transplantation or death.  ***Liver stiffness measurement***  TE examinations were performed before and after HCV treatment in patients with a 3-h fasting by experienced operators. Liver fibrosis staging by TE was considered as reliable for analysis if the following criteria had been met: (1) at least 10 valid measurements; (2) an interquartile range (IQR) lower than 30% of the median of LSM and (3) a success rate of more than 60%. TE examination obtained closest to the date of start and the end of DAA treatment was considered for LSM before treatment and after SVR, respectively. Patients with LSM ≥ 10 kPa before HCV treatment were classified as compensated advanced chronic liver disease (c-ACLD). *l*. Additionally, those patients with LSM ≥ 20 kPa or platelet count < 150 × 10^9^/mm^3^ before HCV treatment were considered as having an unfavorable Baveno VI status.^13^ |
| Bias | 9 | Describe any efforts to address potential sources of bias  **Page 4 (50-53)**  Demographic, clinical and biological data were collected by trained investigators using an electronic case-report form at Research Electronic Data Capture (REDCap, https://projectredcap.org/).  **Page 4 (63-64)**  TE examinations were performed before and after HCV treatment in patients with a 3-h fasting by experienced operators.  **Page 4 (64-66)**  Liver fibrosis staging by TE was considered as reliable for analysis if the following criteria had been met: (1) at least 10 valid measurements; (2) an interquartile range (IQR) lower than 30% of the median of LSM and (3) a success rate of more than 60%. |
| Study size | 10 | Explain how the study size was arrived at  **Flow-chart** |
| Quantitative variables | 11 | Explain how quantitative variables were handled in the analyses. If applicable, describe which groupings were chosen and why  **Page 5 (76-77)**  Continuous variables were reported as median (interquartile range, IQR) and discrete variables were reported as absolute (n) and relative frequency (%). |
| Statistical methods | 12 | (*a*) Describe all statistical methods, including those used to control for confounding  **Page 5 (77-91)**  The duration of follow-up was calculated from the end of treatment to the date of clinical outcome or last visit at one of the two centers until November 30, 2019. If several LRC occurred during the follow-up, the first one was considered for the analysis. The incidence rates of the primary outcomes (per 1,000 person-years) were calculated. The relative risk of incidence of outcomes was evaluated according to surrogate markers of liver dysfunction/fibrosis before HCV treatment and LSM regression post-SVR. A receiver operator characteristic (ROC) curve analysis was performed and the optimal threshold of LSM regression (%) to predict LRC or death was identified using the point nearest to the upper left corner of the ROC curve. Kaplan–Meier curves were plotted and the log-rank test was calculated for univariate analysis. We used the time to event Cox proportional-hazard model to identify factors associated with LRC or death (hazard-ratio, HR) after checking that the main variables verified the proportional-hazard assumption using the Schoenfeld residuals. Variables found to be associated (p value ≤ 0.10) with LRC or death in the univariate analysis were entered into the multivariate Cox models adjusted for age and gender. The variable Baveno VI status (favorable vs. unfavorable) replaced the variables LSM and platelet count at baseline in the models due to collinearity. |
|  |  | (*b*) Describe any methods used to examine subgroups and interactions  **Supplementary Table 1 and 2**  **Page 7 (163-168)**  We observed similar results in a sensitivity analysis considering only patients with unfavorable Baveno VI status before HCV treatment (n=250 and incidence of primary outcomes in 25 patients): low albumin level was independently associated with an increased risk [HR=5.56 (95%CI 2.22-13.97)] and LSM regression after SVR with a decreased risk of LRC or death [HR=0.31 (95%CI 0.12-0.80)] (Supplementary Table 1).  **Page 9 (253-256)**  In the current study, similar results were observed when considering the LSM performed most distantly from the end of treatment [median of 12.5 months (IQR, 10.3-14.9)] instead of the closest TE examination [median of 4.8 months (IQR, 3.4-9.8)] following HCV treatment (Supplementary Table 2). |
|  |  | (*c*) Explain how missing data were addressed  Missing data were declared at the footnote of the tables  **Table 1** Missing data (n): type-2 diabetes (2), hypertension (2), dyslipidemia (9), HCV genotype (1), previous HCV treatment (1), duration of HCV treatment (1), ALT (19), AST (15), total bilirubin (35), albumin (60), INR (68), fasting glucose (34), creatinine (32), platelet count (4).  **Table 2**  ^§^ n=396 patients and 25 clinical outcomes; ^†^ n=452 patients and 28 clinical outcomes. |
|  |  | (*d*) If applicable, explain how loss to follow-up was addressed  Patients with loss of follow-up after SVR were excluded  **Figure 1, flow-chart** |
|  |  | (*e*) Describe any sensitivity analyses  **Supplementary Table 1 and 2**  **Page 7 (163-168)**  We observed similar results in a sensitivity analysis considering only patients with unfavorable Baveno VI status before HCV treatment (n=250 and incidence of primary outcomes in 25 patients): low albumin level was independently associated with an increased risk [HR=5.56 (95%CI 2.22-13.97)] and LSM regression after SVR with a decreased risk of LRC or death [HR=0.31 (95%CI 0.12-0.80)] (Supplementary Table 1).  **Page 9 (253-256)**  In the current study, similar results were observed when considering the LSM performed most distantly from the end of treatment [median of 12.5 months (IQR, 10.3-14.9)] instead of the closest TE examination [median of 4.8 months (IQR, 3.4-9.8)] following HCV treatment (Supplementary Table 2). |
| Results | | |
| Participants | 13* | (a) Report numbers of individuals at each stage of study—eg numbers potentially eligible, examined for eligibility, confirmed eligible, included in the study, completing follow-up, and analysed  **Flow chart**  **Page 5 (96-103)**  A total of 1,131 patients with chronic hepatitis C were treated by DAA in two tertiary centers of Rio de Janeiro (Brazil) from October 2015 to November 2019. One hundred forty-four patients were excluded due to treatment failure (n=26), death before SVR evaluation (n=5), loss of follow-up after SVR (n=87) or missing SVR data (n=26). Furthermore, 120 patients were excluded because of cirrhosis decompensation previously to DAA therapy and 110 patients due to the absence of paired LSM evaluations, before and after HCV treatment. A total of 301 patients were excluded due to LSM < 10 kPa (absence of c-ACLD) before HCV treatment. Figure 1 is a flow chart that summarizes the study enrollment. |
|  |  | (b) Give reasons for non-participation at each stage  **Flow chart** |
|  |  | (c) Consider use of a flow diagram  **Flow chart** |
| Descriptive data | 14* | (a) Give characteristics of study participants (eg demographic, clinical, social) and information on exposures and potential confounders  **Table 1**  **Page 5 (103-108)**  A total of 456 patients [65% female, median age of 62 years (IQR 57-68), median ALT levels of 80 UI/L (IQR, 54-132), 5.5% with HIV coinfection, 88.6% infected by genotype (GT) 1 and 67.5% treated by sofosbuvir/daclatasvir (SOF/DCV)] were included in the study. Additionally, a total of 250 patients [55% (95%CI 51-60)] had an unfavorable Baveno VI status before HCV treatment. Table 1 summarizes demographic and clinical characteristics of patients included in the study. |
|  |  | (b) Indicate number of participants with missing data for each variable of interest  **Page 3 (37)**  Patients with missing data were excluded. |
|  |  | (c) Summarise follow-up time (eg, average and total amount)  **Page 6 (116-118)**  Baseline LSM was performed in a median of 13.7 months (IQR, 7.6-24.7) before the start of HCV treatment by DAA and LSM post-SVR in a median of 4.8 months (IQR, 3.4-9.8) after the conclusion of treatment. During a median follow-up of 2.3 years (IQR, 1.6-2.7) after SVR,(…) |
| Outcome data | 15* | Report numbers of outcome events or summary measures over time  **Page 6 (118-123)**  During a median follow-up of 2.3 years (IQR, 1.6-2.7) after SVR, 28 patients developed a total of 37 primary outcomes (LRC or death): HCC [n=10 (27%), hepatic encephalopathy [n=9 (24%)], ascites [n=9 (24%)], variceal bleeding [n=5 (14%)] or death [n=4 (11%)]. No patient underwent liver transplantation. The overall rate of incidence of clinical outcomes was 29.0 (95%CI 20.0-42.0) per 1,000 person-years. |
| Main results | 16 | (*a*) Give unadjusted estimates and, if applicable, confounder-adjusted estimates and their precision (eg, 95% confidence interval). Make clear which confounders were adjusted for and why they were included  **Pages 6-7 (122-158)**  The overall rate of incidence of clinical outcomes was 29.0 (95%CI 20.0-42.0) per 1,000 person-years. Surrogate markers of liver dysfunction (albumin levels and platelet count) and/or liver fibrosis (LSM) before HCV treatment and LSM regression post-SVR were evaluated to stratify the risk of primary outcomes (LRC or death). Considering the baseline assessment before HCV treatment, the relative risk [RR (95%CI)] of incidence of outcomes was significantly higher in patients with serum albumin levels < 3.5 mg/dL [vs. albumin ≥ 3.5 mg/dL; RR=4.51 (2.06-9.89), p<0.001] and those individuals with unfavorable Baveno VI status [vs. favorable; RR=6.58 (1.99-21.78), p<0.001]. On the other hand, the risk of incidence of outcomes was significantly lower in those patients who had at least 20% regression of LSM post-SVR [19.3 per 1,000 person-years (95%CI 10.7-34.9)] compared with those without [43.0 per 1,000 person-years (95%CI 26.7-69.1)] [RR=0.45 (0.21-0.96), p=0.034] (Table 2). Patients with serum albumin level lower than 3.5 mg/dL before treatment had a higher cumulative incidence of clinical outcomes (95%CI) at 2 years compared with those with albumin ≥ 3.5 mg/dL [17.8% (10.5-29.3) vs 3.6% (2.0-6.7), log-rank p<0.001] (Figure 2A). Additionally, patients with unfavorable Baveno VI status at baseline had significantly higher incidence of LRC or death compared with those with favorable Baveno VI status [9.6% (6.3-14.4) vs 1.1% (0.3-4.2), log-rank p<0.001] (Figure 2B). In contrast, those patients who obtained a regression of at least 20% of LSM post-SVR had significantly lower cumulative incidence of clinical outcomes at 2-year follow-up compared with those without LSM regression [3.4% (1.8-7.0) vs. 9.0% (5.5–14.5), p=0.028] (Figure 3).  **Factors associated with primary outcomes**  The presence of male gender, higher baseline AST levels [per 10 UI/L], higher LSM values, baseline serum albumin < 3.5 mg/dL, baseline platelet count < 150 x 10^9^/mm^3^, presence of baseline unfavorable Baveno VI status and regression of LSM post-SVR were associated (p<0.10) with the incidence of outcomes in the univariate Cox proportional-hazard analysis (Table 3). |
|  |  | (*b*) Report category boundaries when continuous variables were categorized  Category boundaries of continuous variables were described in the results section and Tables/Supplementary Tables  **Table 3 / Supplementary Tables1/2 / Page 7-8 (158-186)**  Those variables were inserted in the multivariate Cox proportional-hazard models adjusted for age and gender. The variables LSM and platelet count before HCV treatment were replaced by the variable “Baveno VI status” in the multivariate models due to strong collinearity. The following factors were independently associated with the incidence of LRC or death after SVR in HCV treated with DAAs [HR (95%CI)]: male gender [vs female, HR=3.00 (1.30-6.95), p=0.010], baseline serum albumin < 3.5 mg/dL [vs ≥ 3.5 mg/dL, HR=4.49 (1.95-10.34), p<0.001] and baseline unfavorable Baveno VI [vs favorable, HR=4.72 (1.32-16.83), p=0.017] (Table 3). In this multivariate model LSM regression of at least 20% after SVR had a trend to reduce the risk of LRC or death [HR=0.45 (95%CI 0.21-1.02), p=0.058]. Additionally, LSM regression post-SVR of at least 20% significantly reduced the risk of LRC or death in multivariate Cox models adjusted for age-and-gender [HR=0.43 (0.20-0.92), p=0.029] and those adjusted for age, gender and baseline albumin level [HR=0.44 (0.20-0.99), p=0.049]. We observed similar results in a sensitivity analysis considering only patients with unfavorable Baveno VI status before HCV treatment (n=250 and incidence of primary outcomes in 25 patients): low albumin level was independently associated with an increased risk [HR=5.56 (95%CI 2.22-13.97)] and LSM regression after SVR with a decreased risk of LRC or death [HR=0.31 (95%CI 0.12-0.80)] (Supplementary Table 1). Moreover, we repeated the analysis considering the LSM more distantly performed from the end of the HCV treatment [median time = 12.5 months (IQR, 10.3-14.9)] to evaluate the impact of LSM regression on incidence of LRC or death. LSM post-SVR remained stable, since the median value (IQR) for the first and the last LSM post-SVR were 12.4 kPa (8.5-19.9) and 12.0 kPa (7.9-19.2), respectively. Male gender [vs female, HR=2.96 (95%CI 1.29-6.82), p=0.011], baseline serum albumin < 3.5 mg/dL [vs ≥ 3.5 mg/dL, HR=4.33 (1.89-9.94), p=0.001], baseline unfavorable Baveno VI [vs favorable, HR=4.72 (1.32-16.8), p=0.017] and at least 20% of LSM regression post-SVR [vs < 20% LSM regression, HR=0.38 (0.17-0.86), p=0.021] were independently associated with the incidence of clinical outcomes after SVR in HCV treated with DAAs in this multivariate analysis (Supplementary Table 2). Regarding the evolution of laboratory parameters, persistent elevation of AST levels after SVR increased the risk of outcomes [HR=10.8 (95%CI 1.30-89.76), p=0.028] and patients with low albumin levels or low platelet count pre-treatment remained at high risk for development of clinical outcomes even if they recovered these parameters after SVR [albumin: HR=3.64 (95%CI 1.43-9.28) and platelet count: HR=3.67 (95%CI 0.92-14.68)]. |
|  |  | (*c*) If relevant, consider translating estimates of relative risk into absolute risk for a meaningful time period  **Page 6 (125-132)**  Considering the baseline assessment before HCV treatment, the relative risk [RR (95%CI)] of incidence of outcomes was significantly higher in patients with serum albumin levels < 3.5 mg/dL [vs. albumin ≥ 3.5 mg/dL; RR=4.51 (2.06-9.89), p<0.001] and those individuals with unfavorable Baveno VI status [vs. favorable; RR=6.58 (1.99-21.78), p<0.001]. On the other hand, the risk of incidence of outcomes was significantly lower in those patients who had at least 20% regression of LSM post-SVR [19.3 per 1,000 person-years (95%CI 10.7-34.9)] compared with those without [43.0 per 1,000 person-years (95%CI 26.7-69.1)] [RR=0.45 (0.21-0.96), p=0.034] (Table 2). |
| Other analyses | 17 | Report other analyses done—eg analyses of subgroups and interactions, and sensitivity analyses  **Supplementary Table 1 and 2**  **Page 7 (163-168)**  We observed similar results in a sensitivity analysis considering only patients with unfavorable Baveno VI status before HCV treatment (n=250 and incidence of primary outcomes in 25 patients): low albumin level was independently associated with an increased risk [HR=5.56 (95%CI 2.22-13.97)] and LSM regression after SVR with a decreased risk of LRC or death [HR=0.31 (95%CI 0.12-0.80)] (Supplementary Table 1).  **Page 9 (253-256)**  In the current study, similar results were observed when considering the LSM performed most distantly from the end of treatment [median of 12.5 months (IQR, 10.3-14.9)] instead of the closest TE examination [median of 4.8 months (IQR, 3.4-9.8)] following HCV treatment (Supplementary Table 2). |
| Discussion | | |
| Key results | 18 | Summarize key results with reference to study objectives  **Page 8 (189-203)**  The current study highlighted that albumin levels lower than 3.5 mg/dL and/or presence of unfavorable Baveno VI status (LSM ≥ 20 kPa or platelet count < 150 x10^9^/mm^3^) before HCV treatment were associated with an increased risk of LRC or death after SVR adjusted for confounding factors in patients with c-ACLD. Additionally, the regression of at least 20% of LSM after SVR was associated with a significant decrease in incidence of LRC or death in patients with c-ACLD treated by DAAs. Our study findings have implications for optimizing management of patients with HCV after SVR. Patients with abnormal liver function tests, low platelet count and/or LSM ≥ 20 kPa before HCV treatment remain at high risk for development of severe outcomes even after SVR. On the other hand, the regression of LSM ≥ 20% during follow-up after HCV eradication seems to be a significant protective factor. The threshold of 20% for LSM regression after SVR compared to before treatment was chosen as the rounded point nearest to the upper left corner of the ROC curve for LSM decrease to predict LRC or death. |
| Limitations | 19 | Discuss limitations of the study, taking into account sources of potential bias or imprecision. Discuss both direction and magnitude of any potential bias  **Page 10 (257-286)**  The main limitation of this study remains the lack of paired liver biopsy to assess the relationship of histological regression of liver fibrosis and lower incidence of clinical outcomes after SVR. However, the accuracy of TE to stage liver fibrosis was extensively validated^24^ and international guidelines have been recommending liver fibrosis staging using LSM by TE in HCV-infected patients.^25^ Additionally, the performance of liver biopsy in SVR patients should be reserved in cases of known or suspected mixed etiologies (e.g. metabolic syndrome, alcoholism or autoimmunity) and for patients who persist with elevated aminotransferases after SVR. We acknowledge that the retrospective study design might be considered a limitation. However, we performed an analysis of a large real-life cohort of patients with HCV treated by DAAs whose medical records for identification of outcomes were reviewed by two trained investigators (JP and CI). We are aware of the relative short follow-up (median time of 2.3 years) in the current analysis. However, in July 2015, first generations of DAAs became available in Brazil and were made available free of charge by the Public Health System (SUS, *Sistema Único de Saúde*), but only for patients with advanced liver fibrosis/cirrhosis. Universal access to DAAs was implemented by the Brazilian Ministry of Health in mid-2017. Nevertheless, our median follow-up time was similar to previous studies and our study identified 37 outcomes after SVR in 28 patients with c-ACLD (LSM ≥ 10 kPa) before HCV treatment. Other potential criticisms might be the absence of sensitivity analyses for factors associated with all-cause mortality or liver neoplasm and the fact that TE examinations before and after SVR were not performed in pre-determined time-points by the same operator. The performance of stratified analyses considering all outcomes or sensitivity time-dependent analyses for single events separately were not feasible due to the relatively small number of clinical outcomes during follow-up. The limited number of events might be explained by the exclusion of patients with previous hepatic decompensation and the relatively short follow-up period. We acknowledge that the 14 months median time from baseline LSM to initiation of DAAs was longer than desirable. However, a period of approximatively 1 year is a relative short time within the natural history of chronic hepatitis C in which significant changes in liver fibrosis might take decades to occur. Additionally, we are aware of the lack of spleen stiffness measurement before and after HCV treatment, the absence of evaluation of portal hypertension progression/regression after SVR and the odds of interobserver variability in liver fibrosis staging using LSM by TE in patients with chronic hepatitis C.^26^ |
| Interpretation | 20 | Give a cautious overall interpretation of results considering objectives, limitations, multiplicity of analyses, results from similar studies, and other relevant evidence  **Pages 8-9 (204-256)**  Large sample size studies, such as the ERCHIVES (n=12,467) and ANRS CO22 Hepather (n=9,895) cohorts, confirmed that interferon-free HCV treatment significantly decreases all-cause mortality.^6,14^ However, the evaluation of the impact of LSM regression after SVR compared with LSM before HCV treatment was not feasible in both studies. Early regression in LSM after SVR might be related to changes in hepatic inflammation rather than fibrosis. However, a large Canadian cohort of HIV-HCV coinfected study that prospectively examined long-term changes in LSM before and after SVR due to DAAs suggested that LSM following SVR likely reflects true reversal of fibrosis.^15^  We acknowledge that the prognostic value of post-SVR LSM remains controversial. Pons *et al* reported that albumin level at baseline and regression of LSM after SVR during follow-up were independently associated with the risk of HCC in 572 patients with LSM ≥ 10 kPa before treatment during a median follow-up of 2.9 years.^16^ In addition, the regression of LSM at SVR compared to 30-days before the start of HCV treatment was related to lower incidence of LRC in the 640 patients from the HEPAVIR Spanish cohort during a median follow-up of 31 months.^17^ However, this study exclusively included HIV-HCV coinfected patients with LSM ≥ 9.5 kPa and the authors did not exclude those patients with prior hepatic decompensation. Semmler *et al* reported that LSM determined after HCV-eradication and its combination with von Willebrand factor antigen and platelet count) could predict post-SVR hepatic decompensation in patients with c-ACLD.^18^ On the other hand, regression of LSM post-SVR were not associated with an increased risk of hepatic decompensation.^19^ However, this study included patients with previous hepatic decompensation and the impact of LSM regression after SVR was evaluated using a lower threshold compared to our study (≥ 20%).  Hepatic venous pressure gradient (HVPG) remains the most robust predictor of clinical decompensation in patients with cirrhosis.^20^ However, HVPG measurement is an invasive method not available worldwide. The role of LSM regression after SVR remains unclear. Despite a markedly decrease in LSM after SVR, this regression seems not to be correlated with HVPG changes in patients with HCV-related cirrhosis and clinically significant portal hypertension before HCV treatment.^21^ On the other hand, Mandorfer *et al* have shown that SVR by DAAs ameliorated portal hypertension and the relative change in LSM (per %; HR=0.972; 95%CI: 0.945-0.999; p=0.044) was a predictor of a HVPG decrease ≥ 10%.^22^ Additionaly, Thabut *et al* analyzed data from 891 patients with HCV with biopsy-proven cirrhosis from the French ANRS CirVir cohort.^23^ The authors reported that unfavorable Baveno VI status at baseline was associated with a significantly lower 5-year overall survival compared to those with favorable Baveno VI at baseline. Moreover, progression of portal hypertension in patients who had HCV suppression at inclusion or during follow-up was more frequent in patients with unfavorable Baveno VI status compared to those with favorable Baveno VI. Our findings reinforced the relationship between the presence of portal hypertension before HCV treatment and clinical outcomes post-SVR: unfavorable Baveno VI status before HCV treatment was associated with a worst prognosis leading to an increased risk of clinical outcomes after SVR [adjusted HR=4.72 (1.32-16.83)] in a multivariate model adjusted for confounding factors. In the current study, similar results were observed when considering the LSM performed most distantly from the end of treatment [median of 12.5 months (IQR, 10.3-14.9)] instead of the closest TE examination [median of 4.8 months (IQR, 3.4-9.8)] following HCV treatment (Supplementary Table 2). |
| Generalisability | 21 | Discuss the generalisability (external validity) of the study results  **Page 11 (300-311)**  (…)the findings of this study showed that low albumin and unfavorable Baveno VI status (LSM ≥ 20 kPa or platelet count < 150 x10^9^/mm^3^) before HCV treatment were associated with an increased risk of clinical outcome during follow-up of patients with c-ACLD. Additionally, our results also suggested a significant decrease in the incidence of LRC or death associated with regression of LSM after SVR in this population. Non-invasive methods are low-cost alternative for monitoring patients with advanced fibrosis/cirrhosis. The use of simple parameters before HCV treatment and repetition of LSM post-SVR can help to identify patients with different risks of developing severe outcome after HCV eradication. |
| Other information | | |
| Funding | 22 | Give the source of funding and the role of the funders for the present study and, if applicable, for the original study on which the present article is based  **Financial support statement**  This work was supported by funding from INI-FIOCRUZ [Programa de Incentivo à Jovens Pesquisadores for HP grant number INI-003-FIO-19-2-5] and from Estacio de Sa University (UNESA) [Programa Pesquisa e Produtividade for GP]. The funders had no role in study design, data collection and analysis, decision to publish or preparation of the manuscript. |

*Give information separately for exposed and unexposed groups.

**Note:** An Explanation and Elaboration article discusses each checklist item and gives methodological background and published examples of transparent reporting. The STROBE checklist is best used in conjunction with this article (freely available on the Web sites of PLoS Medicine at http://www.plosmedicine.org/, Annals of Internal Medicine at http://www.annals.org/, and Epidemiology at http://www.epidem.com/). Information on the STROBE Initiative is available at http://www.strobe-statement.org.
